# Changes in Isthmin-1, Irisin, and the AGE–RAGE Axis Following LSG: A Prospective Single-Center Cohort Study

**DOI:** 10.3390/ijms27198932

**Published:** 2026-10-08

**Authors:** Wojciech Kupczyk, Joanna Boinska, Artur Słomka, Kinga Kupczyk, Marek Jackowski, Ewa Żekanowska

**Affiliations:** 1 Department of General, Gastroenterological, and Oncological Surgery, Faculty of Medicine, Ludwik Rydygier Collegium Medicum in Bydgoszcz, Nicolaus Copernicus University in Toruń, 87-100 Toruń, Poland; 2 Pathophysiology Department, Faculty of Pharmacy, Ludwik Rydygier Collegium Medicum in Bydgoszcz, Nicolaus Copernicus University in Toruń, 87-100 Toruń, Poland; artur.slomka@cm.umk.pl (A.S.);; 3 Department of Hematology and Oncology, National Medical Institute of the Ministry of Interior and Administration, 02-507 Warsaw, Poland

**Keywords:** obesity management, bariatric surgery, isthmin-1, irisin, advanced glycation end products

## Abstract

This prospective single-center cohort study evaluated changes in serum levels of novel adipokines and myokines, including isthmin-1 and irisin, as well as the advanced glycation end product (AGE)–soluble receptor for AGE (sRAGE) axis and monomeric C-reactive protein (mCRP) in patients with obesity before and 5 months after laparoscopic sleeve gastrectomy (LSG). The study included 38 patients with obesity (26 females and 12 males) with a median age of 42 years who underwent LSG. Laboratory parameters were assessed using commercially available immunoenzymatic assays. We observed a significant decrease in isthmin-1 concentration at the postoperative time point (Me = 2.10 ng/mL vs. Me = 1.93 ng/mL, *p* = 0.009). Moreover, a significant increase in irisin levels (Me = 7.33 µg/mL vs. Me = 10.64 µg/mL, *p* = 0.002) and AGEs (Me = 589.50 ng/mL vs. Me = 698.60 ng/mL, *p* = 0.015) was observed after LSG. A significant increase in AGE levels was also noted after normalization to total protein (Me = 8.34 ng/mg protein vs. Me = 10.37 ng/mg protein, *p* = 0.027). sRAGE levels were similar at both time points. Five months after LSG, mCRP levels were significantly lower (Me = 1.99 ng/mL vs. Me = 1.55 ng/mL, *p* = 0.020). Exploratory correlation analyses identified potential associations between AGEs and isthmin-1 (R = 0.37) and between sRAGE and irisin (R = −0.31) preoperatively. Five months after LSG, potential associations were observed between AGEs and irisin (R = 0.38) and between sRAGE and mCRP (R = −0.37). Our findings indicate a decrease in circulating isthmin-1 and mCRP, together with an increase in irisin and AGE concentrations following LSG, suggesting changes in selected metabolic and tissue-related biomarkers after surgery. Further studies are needed to determine the mechanisms underlying these changes and to clarify their potential clinical significance.

## 1. Introduction

Bariatric surgery is currently the most effective treatment for severe obesity, providing sustained weight loss together with substantial improvements in obesity-related metabolic disorders. Although Roux-en-Y gastric bypass (RYGB) has been historically regarded as a well-established bariatric procedure, laparoscopic sleeve gastrectomy (LSG) has become the most frequently performed bariatric procedure worldwide owing to its efficacy, favorable safety profile, and technical simplicity. Numerous clinical studies have demonstrated that both procedures achieve comparable weight loss and metabolic outcomes, although each has distinct advantages [1,2].

The primary therapeutic outcome of bariatric surgery is weight reduction resulting predominantly from the loss of adipose tissue. In absolute terms, patients lose more subcutaneous adipose tissue because it represents the largest fat depot in the body. However, on a relative (%) basis, visceral adipose tissue undergoes a greater reduction than subcutaneous adipose tissue, reflecting its higher metabolic activity [3,4,5]. Indirect effects of bariatric surgery, especially during the early postoperative phase, may also include reductions in lean body mass (primarily due to severe energy restriction, inadequate protein intake, hormonal adaptations, and decreased mechanical loading) and alterations in bone mineral density [6,7].

Bariatric surgery induces metabolic changes that extend far beyond weight loss, including a marked postprandial increase in incretin hormones (i.e., glucagon-like peptide-1), enhanced insulin secretion, improved insulin sensitivity, and rapid and long-term remission of type 2 diabetes in a substantial number of patients [8,9,10]. In addition, reduced systemic inflammation and improved signaling between adipose tissue, muscle, and liver contribute to the restoration of metabolic balance [11,12]. Given the wide range of adaptive mechanisms involved, monitoring the response to bariatric treatment using traditional biochemical parameters appears to be insufficient. Consequently, there is growing interest in identifying novel laboratory biomarkers that better reflect postoperative metabolic remodeling, predict therapeutic outcomes, and enable individualized patient monitoring.

Among these biomarkers, particular attention has recently been directed toward isthmin-1, a newly identified adipokine predominantly secreted by adipocytes. Experimental studies have demonstrated insulin-like properties of isthmin-1, including stimulation of glucose uptake, promotion of protein synthesis, and inhibition of hepatic lipogenesis [13,14]. Interestingly, circulating isthmin-1 concentrations are elevated in obesity and type 2 diabetes. López-Yus et al. further reported that higher isthmin-1 concentrations were associated with a more favorable subcutaneous-to-visceral adipose tissue ratio, suggesting that isthmin-1 may reflect adipose tissue distribution and function rather than overall adiposity [15]. However, evidence regarding changes in circulating isthmin-1 following bariatric surgery remains scarce.

Given the endocrine interplay between adipose tissue and skeletal muscle, irisin has emerged as a potential mediator of the metabolic adaptations following bariatric surgery. Irisin is a myokine involved in energy homeostasis by promoting white adipose tissue browning, thermogenesis, and glucose metabolism. As irisin may also be secreted by adipose tissue, it serves as a mediator of muscle–adipose tissue communication [16,17,18]. However, studies evaluating circulating irisin concentrations after bariatric surgery have presented inconsistent results.

Obesity is also characterized by enhanced oxidative stress and activation of the advanced glycation end product (AGE)–receptor for AGE (RAGE) signaling axis. AGEs accumulate in tissues and the circulation under conditions of chronic inflammation contributing to vascular dysfunction and obesity-related metabolic complications. Binding of AGEs to their receptor (RAGE) amplifies inflammatory and oxidative pathways, whereas soluble RAGE (sRAGE) acts as a decoy receptor, attenuating AGE-mediated signaling [19,20]. Although bariatric surgery improves multiple components of metabolic health, postoperative changes in circulating AGEs and sRAGE remain insufficiently characterized. Further investigation of these mechanisms is of considerable scientific and clinical importance.

Therefore, the aim of the present study was to evaluate changes in circulating concentrations of isthmin-1, irisin, AGEs, soluble RAGE, and monomeric C-reactive protein (mCRP) in patients with severe obesity undergoing LSG. These biomarkers were assessed before surgery and after a 5-month follow-up to evaluate changes following LSG.

## 2. Results

The study cohort included 38 patients who underwent LSG, comprising 26 women and 12 men. Participants were aged between 26 and 64 years, with a median age of 42 years. Associated medical problems were common among the patients, with hypertension (53%), hypothyroidism (24%), and diabetes (16%) being the most frequently observed conditions. The median body mass index (BMI) before surgery was 39.10 kg/m^2^ (IQR: 36.30–44.1), which decreased significantly to 29.95 kg/m^2^ (IQR: 27.60–35.70) five months after LSG (Table 1). A significant reduction in BMI was accompanied by improvements in several body composition parameters, including body fat mass (MBF), percent body fat (PBF), and lean body mass (LBM). In addition, most biochemical markers related to liver and kidney function showed improvement during the postoperative follow-up period, although their values remained within normal reference ranges both before and after surgery, as reported previously [21].

Among the studied inflammatory and metabolic parameters, we observed significant decreases in isthmin-1 and mCRP levels, accompanied by increased concentrations of irisin and AGEs following LSG. The increase in AGE concentrations remained significant after normalization to total protein levels. However, no significant changes over time were observed in sRAGE levels (Table 2).

**Figure 1:**
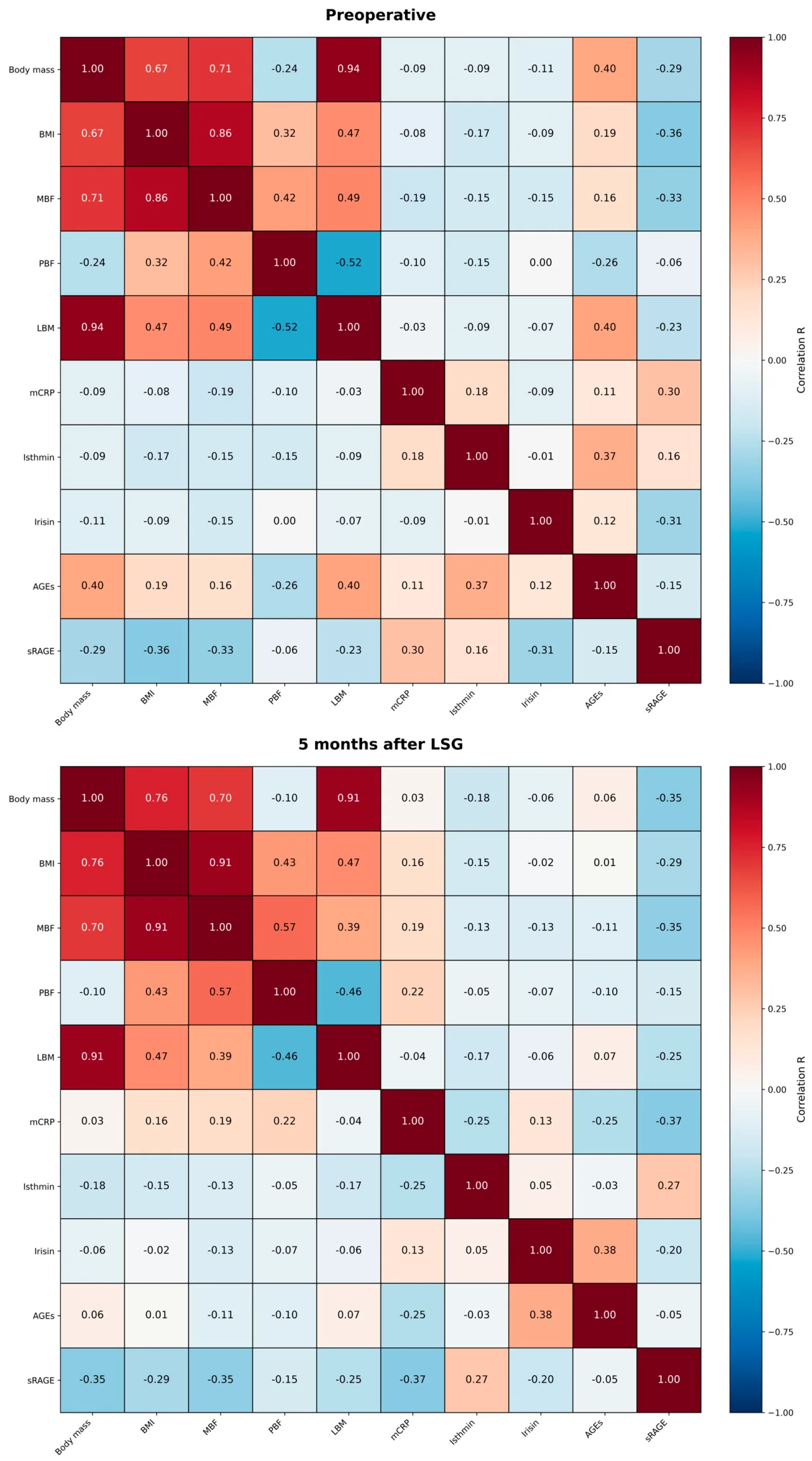
Spearman correlation matrix heat map. Colors indicate Spearman’s correlation coefficients (R).

Exploratory correlation analyses identified potential associations between body composition parameters and adipokines/myokines before and after LSG. As shown in Figure 1, before LSG, AGEs were positively correlated with body mass (R = 0.40), LBM (R = 0.40), and isthmin-1 levels (R = 0.37). In contrast, sRAGE concentrations were negatively correlated with BMI (R = −0.36), MBF (R = −0.33), and irisin levels (R = −0.31). Five months after LSG, AGEs were positively correlated with irisin levels (R = 0.38), whereas sRAGE concentrations were negatively correlated with BMI (R = −0.35), MBF (R = −0.35), and mCRP levels (R = −0.37). Additional exploratory analyses showed similar correlation patterns for AGE/total protein, with correlation coefficients of comparable magnitude and direction to those observed for unnormalized AGE concentrations.

## 3. Discussion

The aim of this study was to investigate adipokine and myokine profiles following LSG. We focused on novel regulatory biomarkers, including isthmin-1, irisin, AGEs, sRAGE, and mCRP, and evaluated their levels before surgery and five months after the bariatric procedure.

The present study demonstrated significant changes in standard anthropometric and body composition parameters. Patients experienced substantial weight loss, reflected by a significant reduction in BMI, together with a marked decrease in adiposity, as evidenced by reductions in both PBF and MBF. Moreover, our study is among the few to demonstrate significant postoperative changes in adipokines and myokines associated with inflammatory status five months after LSG. The observed decrease in isthmin-1 concentrations together with increased irisin levels may reflect changes in adipose tissue- and skeletal muscle-related metabolic signaling following LSG. It is also worth noting that circulating mCRP concentrations significantly decreased following LSG, which may be consistent with attenuation of obesity-associated low-grade chronic inflammation. Furthermore, our study revealed increased circulating AGE concentrations five months after LSG. This increase was observed both for unnormalized AGE concentrations and after normalization to total protein. Despite the increase in AGE concentrations, no significant change in circulating sRAGE levels was observed following LSG. Our study also identified exploratory associations between AGE/sRAGE levels and irisin. In the preoperative period, sRAGE levels were negatively correlated with irisin, whereas five months after LSG, irisin was positively correlated with AGE concentrations. These observations may point to a potential relationship between AGE-related signaling and adipose tissue–skeletal muscle metabolic signaling following LSG; however, they should be interpreted cautiously given the modest strength of the correlations, the exploratory nature of the analyses, and the lack of adjustment for multiple comparisons.

The results of our study are, in several aspects, unique and therefore difficult to compare directly with the available literature. To the best of our knowledge, this is one of the first studies to evaluate circulating isthmin-1 levels before and after LSG. Previous studies have primarily focused on the glucose-regulating properties of isthmin-1 and its circulating levels in patients with diabetes mellitus and related complications such as renal failure [22,23,24]. Lei et al. investigated patients with diabetes mellitus and metabolic dysfunction-associated fatty liver disease (MAFLD). They demonstrated significantly higher circulating isthmin-1 concentrations in patients with diabetes alone and in those with concomitant diabetes and MAFLD compared with healthy controls. Furthermore, they reported a positive correlation between isthmin-1 levels and BMI [25]. This finding is consistent with our observations and supports the hypothesis that obesity, particularly adipose tissue expansion, may contribute to increased isthmin-1 production. Consequently, the marked reduction in adipose tissue following LSG could explain the postoperative decrease in circulating isthmin-1 observed in our study. In contrast to our findings, a recently published study by Ag O. et al. (2026) did not reveal any significant changes in isthmin-1 levels three months after LSG, regardless of the magnitude of weight loss [26]. The discrepancy between our study and theirs may be explained by the different follow-up periods (3 months vs. 5 months), which could have significantly influenced the observed results.

López-Yus et al. suggested that higher circulating isthmin-1 levels may be associated with a more favorable body fat distribution, particularly a higher subcutaneous-to-visceral adipose tissue ratio [15]. In addition, experimental studies have suggested a potentially beneficial role of isthmin-1 in glucose regulation and insulin sensitivity [13,14]. However, clinical studies have reported associations between higher circulating isthmin-1 levels and renal abnormalities in patients with type 2 diabetes, including greater albuminuria and lower eGFR [23,24]. Therefore, the decrease in isthmin-1 observed after LSG cannot be unequivocally interpreted as a beneficial metabolic change, and its clinical significance requires further investigation.

The present study revealed a significant increase in irisin levels five months after LSG. However, the available literature on this topic is inconsistent and only partially supports our findings. A study by Kumar et al. (2019) [27] reported increased irisin levels in adolescents at both 6 and 12 months after bariatric surgery. Moreover, the authors found negative correlations between changes in irisin levels and changes in BMI (r = −0.64, *p* = 0.04), as well as between changes in irisin and changes in interleukin-6 levels (r = −0.67, *p* = 0.01) [27]. Similarly, Gluck et al. (2019) observed increased irisin concentrations six months after LSG [28]. Both studies suggested that the reduction in adipose tissue following bariatric surgery may promote a shift toward increased myokine production, which could contribute to the observed rise in circulating irisin levels. In contrast, studies by Yin J. et al. (2023) and Moehlecke M. et al. (2025) reported a decrease in irisin levels at 3 and 6 months after bariatric surgery, respectively [29,30]. According to these authors, the reduction in adipose tissue following LSG or RYGB may account for the decrease in irisin levels, as adipose tissue is one of the sources of this myokine. Similarly, Karam R.A. et al. (2024) [31] observed decreased irisin levels 12 months after LSG. They also reported significant negative correlations between irisin levels and low-density lipoprotein (LDL) cholesterol, triglycerides, C-reactive protein (CRP), and high-sensitivity cardiac troponin [31]. Based on these findings, the authors suggested that irisin may serve as a biomarker of cardiovascular risk. If lower irisin levels are indeed associated with a less favorable cardiovascular profile, the increase in irisin observed after LSG in our study may be consistent with improved cardiac health. However, this interpretation remains hypothetical and requires confirmation in further prospective studies. In addition, comparisons of irisin concentrations across studies should be made with caution, as different ELISA-based assays and manufacturers may differ in antibody specificity and analytical characteristics. Previous studies have raised concerns regarding the specificity of some commercially available irisin assays [32]; however, the assay used in the present study showed no cross-reactivity with a broad panel of potentially interfering human proteins, according to the manufacturer’s validation data. Nevertheless, differences between assays may contribute to variability in irisin measurements and should be considered when interpreting the findings and drawing conclusions regarding changes in circulating irisin levels.

Our study also demonstrated a significant increase in circulating AGE levels five months after LSG, both for unnormalized AGE concentrations and after normalization to total protein, while no significant changes were observed in sRAGE concentrations. The available literature on this topic is limited, and the existing findings are inconsistent. Sánchez et al. proposed that AGEs accumulate in tissues over many years; therefore, their increased release into the circulation following bariatric surgery may reflect the mobilization of long-term tissue deposits rather than enhanced endogenous AGE formation [33]. Thus, an increase in circulating AGE concentrations does not necessarily imply an increase in the total AGE burden, but may instead reflect a shift in AGEs from tissue stores into the circulating pool. In contrast, Deo et al. reported a reduction in AGE levels following weight loss induced by an energy-restricted diet [19]. The discrepancy between these findings may be explained by differences in the study populations, weight-loss interventions, timing of sample collection, and analytical methods used to assess AGEs.

The increase in circulating AGE concentrations observed in our study should be interpreted in the context of several factors. On the one hand, patients undergoing LSG receive dietary guidance and experience substantial changes in both the quantity and quality of their diet. Given the reduced food intake and generally lower consumption of highly processed foods after surgery, exposure to dietary AGEs would be expected to decrease, although dietary AGE intake was not directly assessed in our study. On the other hand, as reflected by our bioimpedance measurements, weight loss following LSG was accompanied not only by a reduction in body mass but also by changes in ECW and ICW compartments, which may affect the circulating concentrations of proteins and other plasma constituents. Finally, the ELISA used in our study measures total circulating AGEs without distinguishing individual AGE species or their protein substrates. According to the manufacturer’s data, no significant cross-reactivity or interference was observed with the tested AGE analogues; however, the assay does not provide information on the specific chemical composition of the measured AGE pool. Therefore, the observed increase in circulating AGEs should be interpreted cautiously and cannot be attributed to a single underlying mechanism.

In the present study, no significant changes in sRAGE concentrations were observed after LSG. Similarly, Kanikowska et al. did not detect significant changes in sRAGE levels following an eight-week calorie-restricted diet [34]. In contrast, several studies have reported increased sRAGE concentrations 12 and 24 months after bariatric surgery [35,36]. These observations suggest that changes in sRAGE may occur later during the postoperative period and may not be detectable during the early phase of rapid weight loss. The lack of a corresponding increase in sRAGE may also be influenced by methodological limitations. Commercial ELISA assays quantify total circulating sRAGE but generally do not distinguish between ligand-free and ligand-bound forms. Consequently, the measured sRAGE concentration may not accurately reflect its biological availability or functional capacity to neutralize circulating AGEs.

A weak positive correlation between postoperative AGE and irisin levels was observed after LSG. This association may be related to metabolic adaptations accompanying weight loss, although other factors, including changes in adipose tissue metabolism or AGE turnover, may also contribute. However, the biological significance of this association remains unclear, and the finding should be considered hypothesis-generating.

To our knowledge, clinical data evaluating circulating mCRP in patients undergoing bariatric surgery are currently lacking. mCRP, the biologically active pro-inflammatory isoform, has been investigated mainly in experimental settings and in the context of obesity-related inflammation [37,38]. The results of our study demonstrated that LSG was associated with weight loss, while the reduction in adiposity was accompanied by a decrease in the inflammatory marker assessed.

The presented study has several limitations. First, the relatively small sample size, the short follow-up period of 5 months, and the single-center design may limit the generalizability of the findings. In addition, the relatively small sample size may have limited the statistical power to detect smaller effect sizes. In addition, the study was conducted in a predominantly Caucasian population, which may further limit the applicability of the results to other ethnic groups. The single-arm design and the lack of a non-surgical or diet-matched comparator preclude direct comparison with alternative approaches and limit the ability to distinguish changes specifically attributable to LSG from those related to other factors. Second, the absence of a broader panel of biochemical and metabolic parameters limits the possibility of relating the observed changes in adipokines, myokines, AGEs/sRAGE, and mCRP to other metabolic and inflammatory pathways. Moreover, the relatively small changes observed in some biomarkers may require more sensitive and specific analytical methods, such as mass spectrometry-based approaches, to allow more precise characterization and identification of individual analytes. In particular, the use of commercial ELISA-based assays should be considered when interpreting the absolute concentrations of the analyzed biomarkers, as values obtained using different assay platforms may not be directly comparable. Furthermore, several clinical and behavioral factors, including smoking status and associated medical problems, may have acted as potential confounders and could have influenced the observed biomarker changes. For this reason, future studies including larger and more diverse populations and appropriate comparator groups should be considered to better characterize the observed associations and investigate their potential clinical relevance.

Despite these limitations, the exploratory nature of the present study provides hypothesis-generating observations regarding changes in circulating biomarkers following LSG and may serve as a basis for further studies investigating the complex relationships between adipose tissue, skeletal muscle, and inflammatory pathways.

Taken together, our findings indicate a decrease in circulating isthmin-1 and mCRP, together with an increase in irisin and AGE concentrations following LSG, suggesting changes in selected metabolic and tissue-related biomarkers after surgery. Further studies are needed to determine the mechanisms underlying these changes and to clarify their potential clinical significance.

## 4. Materials and Methods

### 4.1. Study Group

**Figure 2:**
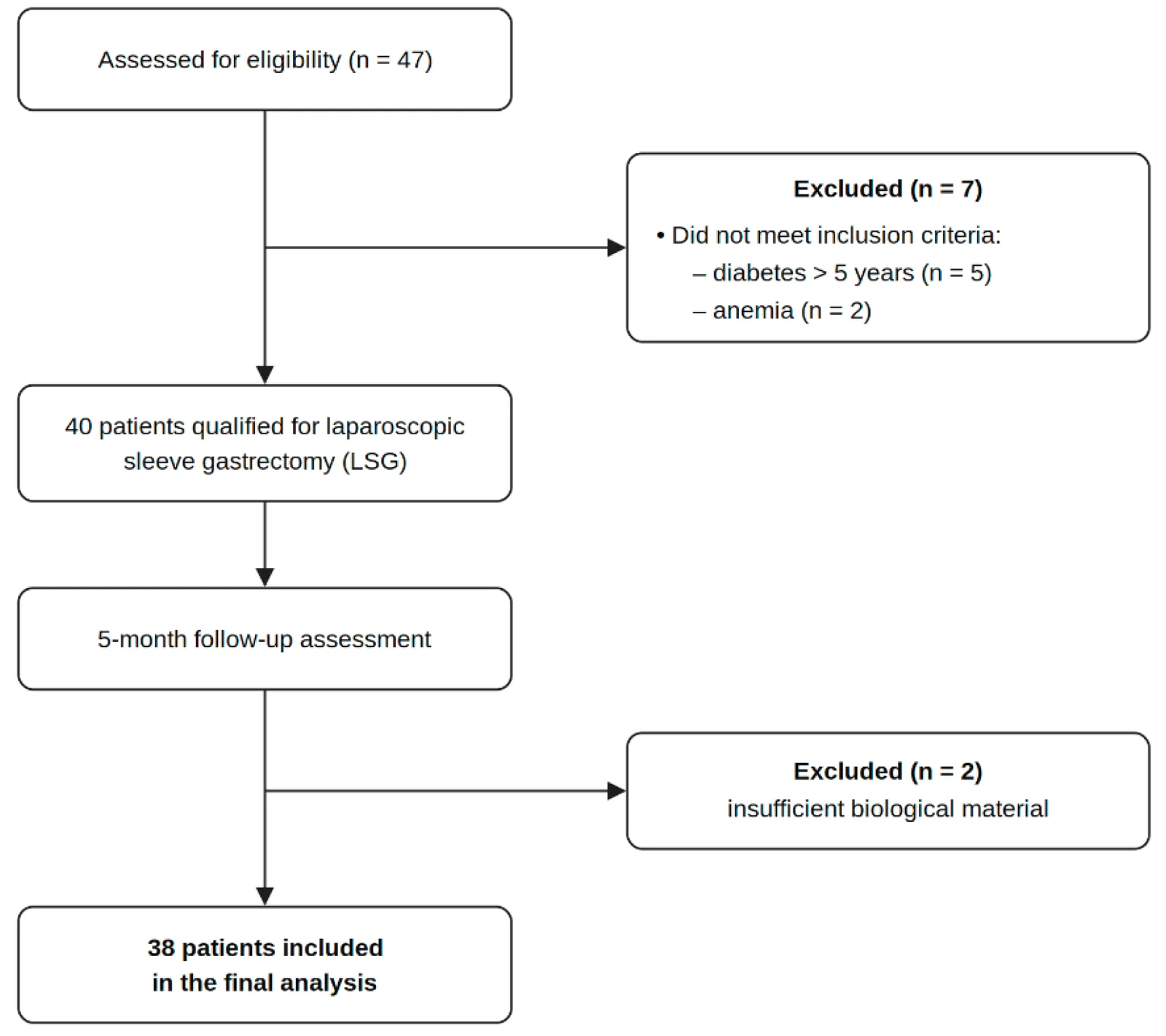
Flow diagram of patient selection.

This was a prospective, single-center cohort study. The study enrolled 38 patients with obesity who underwent LSG at the Department of General, Gastroenterological and Oncological Surgery, Collegium Medicum, Nicolaus Copernicus University in Toruń, Poland, between 2022 and 2023 (Figure 2). All 38 patients completed the 5-month follow-up, and complete data were available for all participants, with no dropouts during the follow-up period. The present study was conducted in a subgroup of a previously described cohort of patients undergoing LSG at our center [21].

Eligibility for bariatric surgery was established according to the recommendations of the European Association for Endoscopic Surgery published in 2020 [39]. Patients qualified for surgery if they presented with a BMI ≥ 40 kg/m^2^ or a BMI between 35 and 40 kg/m^2^ accompanied by associated medical problems likely to improve after weight reduction.

All participants received comprehensive nutritional counseling during the preoperative, perioperative, and postoperative periods, together with long-term postoperative follow-up. Exclusion criteria included previous bariatric procedures, severe cardiovascular or pulmonary diseases, active malignancy treatment, alcohol or substance abuse, significant psychiatric disorders, anemia, diabetes mellitus lasting longer than five years, and current iron supplementation.

Venous blood samples for biochemical analyses were collected one day before surgery and again five months postoperatively during routine follow-up visits. Anthropometric measurements were performed at the same time points. Written informed consent was obtained from all participants prior to inclusion in the study. The study protocol was approved by the Bioethics Committee of Nicolaus Copernicus University in Toruń (KB 82/2021).

### 4.2. Body Composition

Body weight and height were measured in all participants. Body mass index (BMI) was calculated using the standard formula: weight (kg)/height (m^2^). In addition, body composition was assessed using a body composition analyzer (X-Contact 350, Jawon Medical, Seongnam, Republic of Korea). The assessed parameters included body fat mass (MBF), percent body fat (PBF), lean body mass (LBM), extracellular water (ECW), intracellular water (ICW), and excess body weight (EW).

### 4.3. Biochemical Parameters

Venous blood samples were obtained at baseline and again after a 5-month follow-up period in all 38 participants, with complete blood sample data available for all participants. Blood was collected into Vacutainer tubes (BD Biosciences, San Jose, CA, USA) containing either K2EDTA or a clot activator. The samples were centrifuged at 2500× *g* for 20 min at 4 °C, after which plasma and serum were separated and stored at −80 °C until biochemical analyses were performed. Baseline and 5-month samples from each patient were analyzed using the same ELISA assay and the same reagent set.

The serum irisin levels were measured by IRISIN ELISA (RAG018R, BioVendor R&D, Brno, Czech Republic). The manufacturer reported intra-assay CVs of 4.8–8.1% and inter-assay CVs of 8.0–9.7%, and a limit of detection (LOD) of 1 ng/mL. Plasma isthmin-1 (ISM-1) levels were assessed using the ELISA kit by BioVendor R&D (RAG031R, BioVendor R&D, Brno, Czech Republic). The assay had a limit of detection of 0.4 ng/mL, with an intra-assay coefficient of variation of 4.3–6.6% and an inter-assay coefficient of variation of 2.3–8.5%. Serum advanced glycation end product (AGE) concentration was determined using ELISA kit (CEB353Ge, Cloud-Clone Corp., Katy, TX, USA). According to the manufacturer, the assay showed intra-assay and inter-assay coefficients of variation below 10% and 12%, respectively, with a minimum detectable dose of less than 38.2 ng/mL. Soluble receptor for advanced glycation end product (sRAGE) levels were measured using ELISA technique (RD1911162R, BioVendor R&D, Brno, Czech Republic). The assay had a limit of detection of 19.2 pg/mL, with an intra-assay coefficient of variation of 2.6–5.3% and an inter-assay coefficient of variation of 5.5–8.8%. Monomeric C-reactive protein (mCRP) concentrations were determined using HUMAN MONOMERIC CRP ELISA (RBL010R, BioVendor R&D, Brno, Czech Republic). The assay had a limit of detection of 0.63 ng/mL, with an intra-assay coefficient of variation of 4.3–8.1% and an inter-assay coefficient of variation of 5.0–14.5%.

All laboratory analyses, except for total protein, were performed in the Department of Pathophysiology laboratory in accordance with the manufacturers’ instructions. Total protein concentration was measured at the hospital during the procedure and at follow-up using a Beckman Coulter AU680 automated chemistry analyzer (Beckman Coulter, Inc., Brea, CA, USA), according to the manufacturer’s instructions.

### 4.4. Statistical Analysis

Statistical analyses were performed using Statistica version 13 (TIBCO Software Inc., Palo Alto, CA, USA). No a priori sample size calculation was performed for this study. The Shapiro–Wilk test was used to assess the distribution of continuous variables. As the data were not normally distributed, continuous variables are presented as medians with interquartile ranges (IQRs). Within-group changes between baseline and follow-up were evaluated using the Wilcoxon signed-rank test. For comparisons of variables over time, the Hodges–Lehmann estimator of the paired difference (postoperative minus preoperative values) was calculated, together with the corresponding 95% confidence intervals (CIs). Correlations were assessed using Spearman’s rank correlation test. Correlation analyses were exploratory and unadjusted, and no correction for multiple comparisons was applied. Missing data were not imputed, and analyses were performed using the available data. AGE concentrations were normalized to total protein content and expressed as ng/mg protein, according to the following formula: AGEs (ng/mL)/[total protein (g/dL) × 10]. A two-sided *p*-value of <0.05 was considered statistically significant.

## Figures and Tables

**Table 1:** Clinical characteristics.

Characteristics	Median (IQR)	Range	*p* Value
Age, years	42 (33–45)	26–64	-
Height, m	1.72 (1.63–1.79)	1.50–1.96	-
Weight, kg			
Baseline	114.35 (103.40–133.80)	88.30–185.80	<0.0001
At 5 months	88.30 (78.00–108.50)	66.50–171.40	
BMI, kg/m^2^			
Baseline	39.10 (36.30–44.10)	35.60–49.10	<0.0001
At 5 months	29.95 (27.60–35.70)	23.80–44.60	
MBF, kg			
Baseline	46.70 (41.60–52.70)	27.40–71.00	<0.0001
At 5 months	29.00 (25.10–37.40)	17.60–53.90	
PBF, %			
Baseline	42.55 (34.80–44.70)	27.80–49.00	<0.0001
At 5 months	33.45 (28.80–38.20)	19.70–44.40	
LBM, kg			
Baseline	64.40 (59.50–83.60)	49.30–118.30	<0.0001
At 5 months	55.70 (51.80–76.00)	42.60–122.80	
ECW, kg			
Baseline	19.05 (17.40–24.60)	14.30–34.80	<0.0001
At 5 months	16.40 (15.30–22.40)	12.40–36.30	
ICW, kg			
Baseline	27.60 (25.50–35.90)	21.20–50.40	<0.0001
At 5 months	23.90 (22.30–32.30)	18.30–52.10	
EW, kg			
Baseline	48.95 (39.80–64.90)	21.20–97.90	<0.0001
At 5 months	22.80 (16.30–38.30)	5.10–86.90	

Abbreviations: BMI, body mass index; ECW, extracellular water; EW, excess weight; ICW, intracellular water; LBM, lean body mass; MBF, mass of body fat; PBF, percent body fat.

**Table 2:** Pre- and postoperative metabolic-inflammatory markers in bariatric surgery patients.

Variable	Preoperative n = 38	5 Months After LSG n = 38	Hodges–Lehmann Estimate	95%CI	*p*-Value
Isthmin-1, ng/mL	2.10 (1.60–3.86)	1.93 (1.57–2.45)	−0.30	(−0.75, −0.07)	0.009
Irisin, µg/mL	7.33 (5.33–10.94)	10.64 (7.38–14.53)	2.86	(1.07, 4.95)	0.002
AGEs, ng/mL	589.50 (466.10–687.60)	698.60 (588.40–795.40)	97.70	(19.40, 179.10)	0.015
AGE/total protein, ng/mg protein	8.34 (6.37–9.59)	10.37 (8.57–11.66)	1.59	(0.49–2.84)	0.027
sRAGE, pg/mL	630.90 (492.90–1002.00)	705.75 (521.10–922.50)	36.25	(−62.15, 135.45)	0.460
mCRP, ng/mL	1.99 (1.37–4.77)	1.55 (0.82–2.65)	−0.88	(−2.20, −0.22)	0.020

Hodges–Lehmann (HL) estimator; CI, confidence interval. The HL estimator represents the estimated median treatment effect based on the paired differences (postoperative minus preoperative values). Abbreviations: AGEs, advanced glycation end products; sRAGE, soluble receptor for advanced glycation end products; mCRP, monomeric C-reactive protein; LSG, laparoscopic sleeve gastrectomy.

## Data Availability

The original contributions presented in this study are included in the article. Further inquiries can be directed to the corresponding author.
